# Effect of Spray-Type Alginate Hydrogel Dressing on Burn Wounds

**DOI:** 10.3390/gels10020152

**Published:** 2024-02-19

**Authors:** Jeong Yeon Choi, Yong-Joon Joo, Ri Jin Kang, Hee Kyung Jeon, Gyeong Sik Hong

**Affiliations:** 1 Advanced Mechatronics R&D Group, Daegyeong Regional Division, Korea Institute of Industrial Technology (KITECH), 320 Technosunhwan-ro, Yuga-eup, Dalseong-gun, Daegu 42994, Republic of Korea; jychoi77@kitech.re.kr; 2 INCORE Co., Ltd., Hyeoksin-daero 78-gil, Dong-gu, Daegu 41070, Republic of Korea; 3 Department of Pharmacology, School of Medicine, Kyungpook National University, Daegu 41918, Republic of Korea; 4 Advanced Energy Materials and Components R&D Group, Korea Institute of Industrial Technology (KITECH), 42-7, Baegyang-daero 804 beon-gil, Sasang-gu, Busan 46938, Republic of Korea; jeonhk75@kitech.re.kr

**Keywords:** spray type, alginate, hydrogel, burn, wound healing

## Abstract

Immediate burn wound care is a critical factor influencing the outcomes of burn treatment. In this study, we developed a spray-type alginate hydrogel dressing that promotes wound healing, reduces pain, and increases the convenience of use in a burn treatment emergency. We investigated the efficiency of newly developed spray-type alginate hydrogel dressing on the wound healing process. We investigated the efficacy of the alginate hydrogel dressing for wound healing in 30 Sprague Dawley rats. Four deep, round second-degree burn wounds (diameter, 1.5 cm) were created bilaterally on the dorsum of the rat’s trunk; the rats were divided into four groups, in which different dressing materials were applied as follows: group A, gauze (control); group B, Mepilex™ (control); group C, 2.25% alginate hydrogel; and group D, 2.5% alginate hydrogel. The gross findings of each group were compared by tracing the remaining wound and performing visual and histological observations and biochemical analysis for proteins associated with wound healing at each time period. In burn wounds, groups C and D showed significantly higher contraction, epithelialization, and healing rates. Histologically, groups C and D showed an improved arrangement of collagen fibers and a thick epithelial layer 14 days after initial wound formation. Group C showed higher CD31, TGF-β, and fibronectin expression in Western blot analyses after day 14. This study suggests that the spray-type alginate hydrogel dressing is an effective material for initial burn wound care.

## 1. Introduction

**Figure 1:**
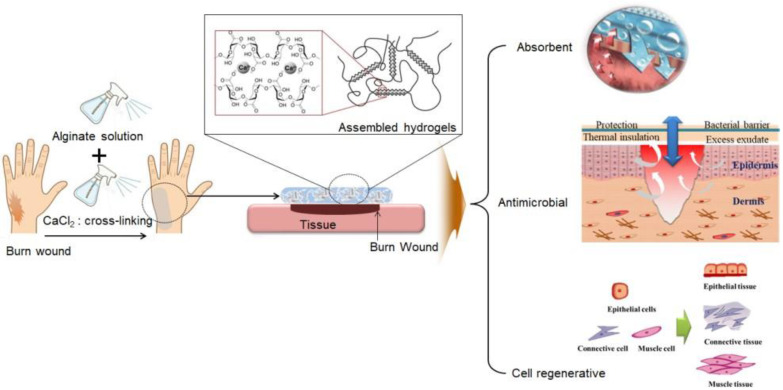
Concept of burn wound healing using a spray-type alginate hydrogel dressing.

The skin performs various functions essential for the survival of the human body. Representative (typical) skin functions include creating a barrier to maintain homeostasis inside the human body in response to environmental changes, a sensory function to recognize external changes, and temperature-control functions (sweating). However, when the skin is damaged due to burns or trauma, its protective function is lost [1,2,3]. Burns are diseases in which skin cells are destroyed or necrosed by heat, damaging not only the skin but also the skin appendages (hair follicles, sweat glands, and sebaceous glands). When the skin is damaged, various secondary diseases appear along with infection. Inflammation and extreme pain are serious systemic conditions that can lead to death. Burns are divided into first-, second-, and third-degree, depending on the symptoms. First-degree burns affect the upper skin layers (epidermis), second-degree burns involve the lower skin layer (dermis), and third-degree burns affect a large area (superficial–partial thickness burn), and the deep layers of the skin (subcutaneous tissue) are damaged [4,5]. Burns are easy to recognize owing to the symptoms and burn areas, or areas with good prognosis, but treatment is difficult, especially in areas on the body with curves such as the armpits, groin, and anus. Nevertheless, research on the physiological mechanisms of burns and drug development for effective treatment is insufficient [6,7]. The best burn treatments prioritize lowering the temperature of the wounded area (cooling), suppressing the occurrence of additional infections, and preventing wounds from drying [8]. Gauze was commonly used to heal burns, but wound dressings are now used. Wound dressings protect the wounds, prevent contamination, and prevent the absorption of exudate, bleeding, and the loss of body fluids. However, when wound dressings are used on curved areas of the body, adhesion occurs between the affected areas, and when applied to a wide area, the probability of skin loss or secondary infection increases due to moisture evaporation, which limits its use [9,10,11]. Thus, there is an urgent need to develop functional materials with excellent biocompatibility that can be used to treat a wide range of burns on normal or curved areas of the body, minimize side effects, improve blood circulation, and alleviate skin and tissue necrosis [12]. Recently, interest in hydrogels as formulations for various materials has increased. Hydrogels are three-dimensional hydrophilic polymer structures that contain large amounts of moisture and can absorb at least 20% of their total weight. Additionally, they can be manufactured into various forms and properties depending on the chemical structure of the hydrophilic polymer used or the degree of cross-linking between the polymer chains. However, its application on large or curved areas without physical contact is limited [13,14,15,16]. Examples of materials used in hydrogels include poly-2-hydroxy-ethyl methacrylate (PHEMA) and alginic acid, which are highly hydrophilic and biocompatible [17]. Particularly, alginic acid is a natural polysaccharide composed of β-D mannuronic acid and R-L guluronic acid units extracted from the cell walls of marine brown algae and bacteria, which form hydrogels by ionic bonding with divalent cations such as Ca^2+^. It effectively absorbs the exudate generated when a wound forms and is a natural polymer that is widely applied in pharmaceutical and medical devices owing to its biocompatibility and non-toxicity [18]. Studies on wound dressings that combine active substances with various natural materials, such as glucuronic acid, aloe vera, hydroxylated lecithin, lysozyme, hyaluronic acid, and vicenin-2 based on alginic acid, are actively being conducted. Wound dressing preparations using alginic acid include films, hydrogels, fibers, and foams manufactured to suit the wound [19,20,21]. However, these materials are difficult to modify in advance, can only be fabricated to a certain size, and require an adhesive coating layer for fixation, which limits their use. Therefore, in this study, we developed a spray-type alginate hydrogel dressing for emergency burn treatment that can promote wound healing, reduce pain, and is user-friendly. The stability of the developed material was reported in a previous paper [22]. To use the developed hydrogel dressing as an effective treatment for skin defects, it applied in the form of a spray to cover the surface of the burn wounds and prevent primary infection (Figure 1). In addition, the efficacy of the newly developed dressing in the wound-healing process was evaluated using a rat model.

## 2. Results and Discussion

### 2.1. Evaluation of the Stability of a Gel Formulation

**Figure 2:**
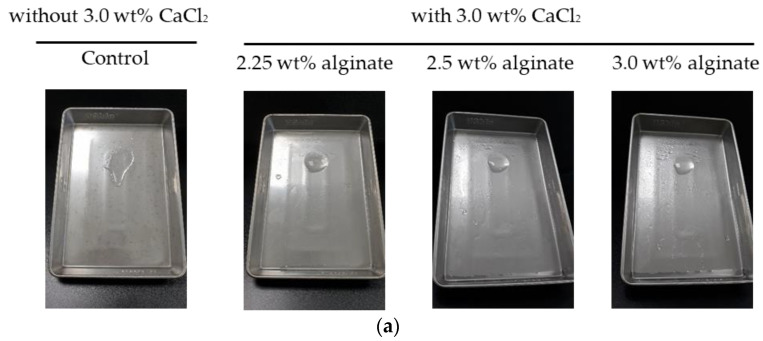

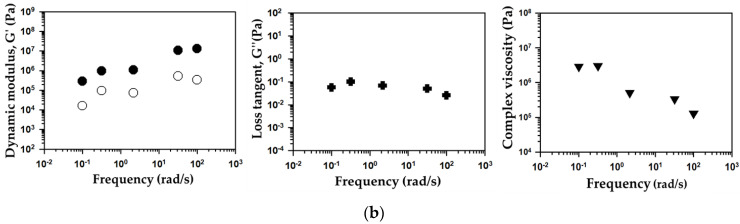
Analysis of stability of a gel formulation: (**a**) gelation time, (**b**) viscoelastic evaluation using rheometer of 2.5 wt% alginate solution with 3.0 wt% CaCl_2_ solution group.

It was possible to spray smoothly in all groups, and the concentration of the cross-linking agent (CaCl_2_) was expressed at 3.0 wt% due to the problem of flowing down after spraying. As a result of conducting a gel-maintenance experiment, it was confirmed that the gel was not maintained without a cross-linking agent, and when a cross-linking agent was sprayed, it was confirmed that the gel was maintained for more than 6 h in all groups (2.25, 2.5, and 3.0 wt% alginate solution) under wet surface conditions (Figure 2a).

Particularly, as a result of conducting a viscoelastic behavior experiment using a rheometer of the 2.5 wt% alginate solution with 3.0 wt% CaCl_2_ solution group that looked stable with the spray and gel membrane, it was confirmed that G’ > G” was shown in the entire frequency region, indicating typical rheological behavior of the gel. The dynamic modulus (G’) and loss tangent (G”) at a frequency of 0.1 rad/s were 0.29 and 0.016 MPa, respectively, and the complex viscosity was measured at 2.88 MPa. Loss tangent and complex viscosity tended to decrease with frequency, confirming the behavior of composite fluids mixed with aqueous solutions in addition to cross-linked gels (Figure 2b).

### 2.2. In Vivo Evaluation

The healing effect was evaluated by applying the gel formulation to the burn animal model using the 2.25 wt% alginate solution with 3.0 wt% CaCl_2_ solution group and 2.5 wt% alginate solution with 3.0 wt% CaCl_2_ solution group, which showed stable gelation performance.

#### 2.2.1. Visual Evaluation

**Figure 3:**
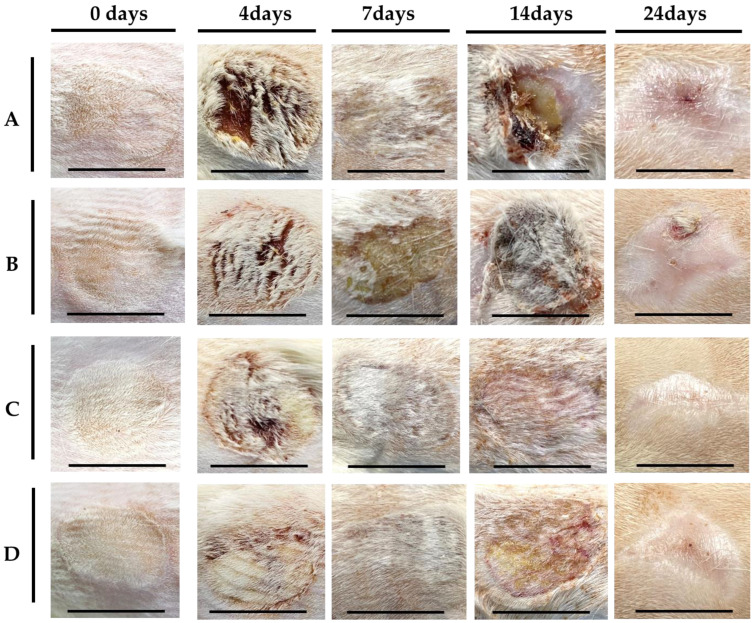
Photographs of visual evaluation (scale bar; diameter of 2.5 cm).

**Figure 4:**
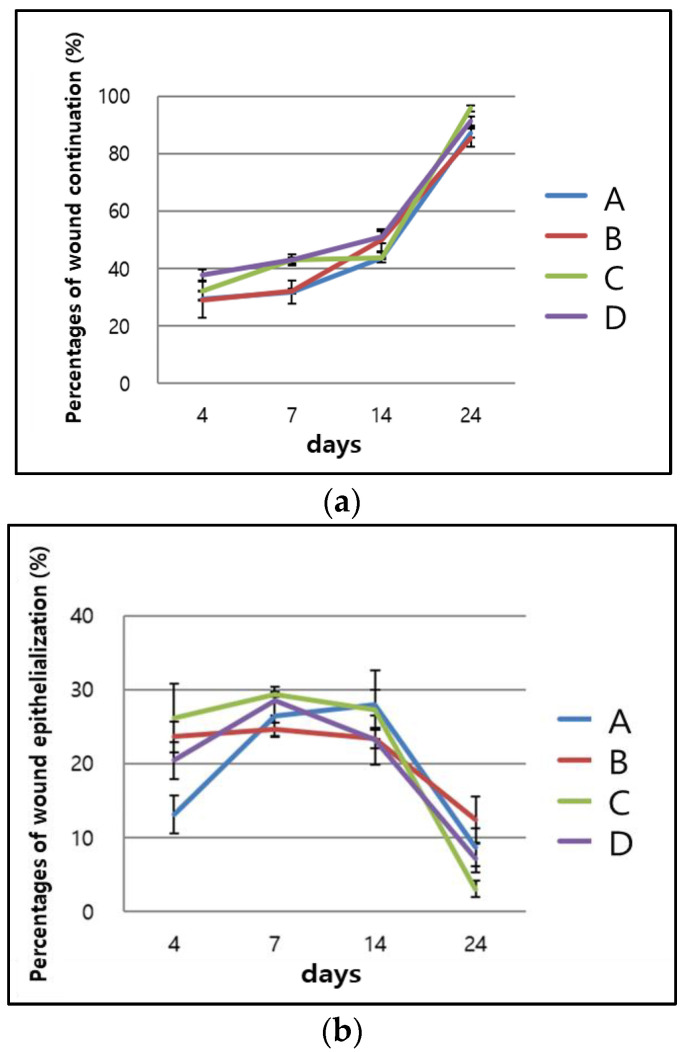

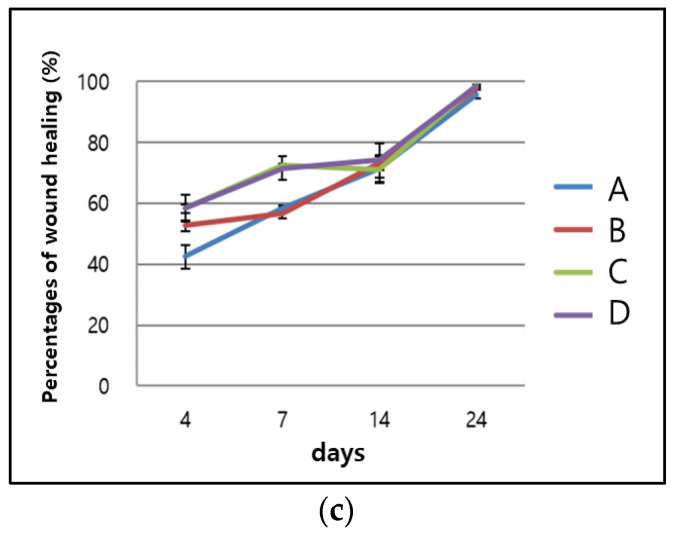
Wound contraction (**a**), wound epithelization (**b**), and wound healing rate (**c**) for A. Gauze, B. Mepilex Lite, C. 2.25 wt% alginate solution with 3.0 wt% CaCl_2_ solution, D. 2.5 wt% alginate solution with 3.0 wt% CaCl_2_ solution.

After the burn wound was induced, exudates and scars were observed (scale bar; diameter 2.5 cm). Over time, after the scar was removed, the wound healed as the burn contracted, and fibrotic granulation tissue decreased. In all groups, the scar was observed up to 4 days after wounding, and after the eschar fell off, it was observed that it shriveled and epithelialized over time, resulting in a gradual decrease in micro-epithelial granulation tissue. No signs of infection were observed in any of the groups (Figure 3). The wound contraction rate was higher on day 14 in groups C and D than in the control groups (A and B), and the wound healing rate was more than 10% faster than that in the control groups on day 7. Groups C and D showed significantly higher contraction, epithelization, and healing rates (Figure 4).

#### 2.2.2. Histological Evaluation

**Figure 5:**
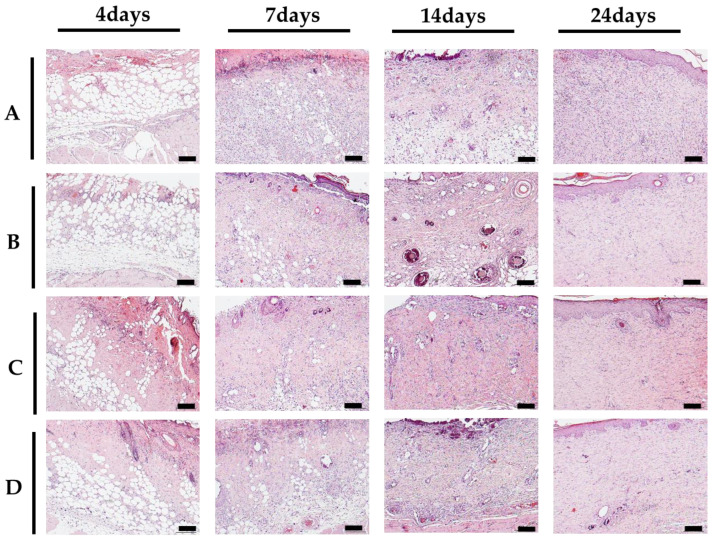

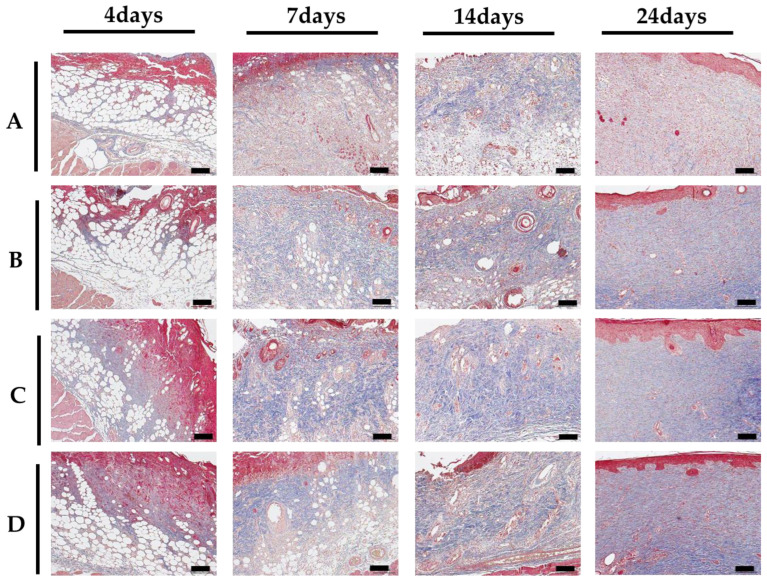
Histologic findings of each group ((**above**): H&E, (**below**): Masson’s trichrome, 10× magnification, scale bar of 500 μm): for A. Gauze, B. Mepilex Lite, C. 2.25 wt% alginate solution + 3.0 wt% CaCl_2_ solution, D. 2.5 wt% alginate solution + 3.0 wt% CaCl_2_ solution.

On day 7 after burn induction, full-thickness skin defect exudates and inflammatory cell infiltration, such as polynuclear cells and lymphocytes, were observed in all groups (Figure 5 above). On day 14, neovascularization was increased in groups C and D compared to the control groups, and on day 24, groups C and D showed better growth than the control groups (Figure 5). On days 7 and 14, more collagen fibers were formed in groups B, C, and D than in group A, and the density of the collagen fibers was normally arranged in groups C and D, especially on day 24 (Figure 5 below). Histologically, groups C and D showed a better arrangement of collagen fibers and thick epithelial layer on the 14th day after wound formation.

#### 2.2.3. Biochemical Evaluation

**Figure 6:**
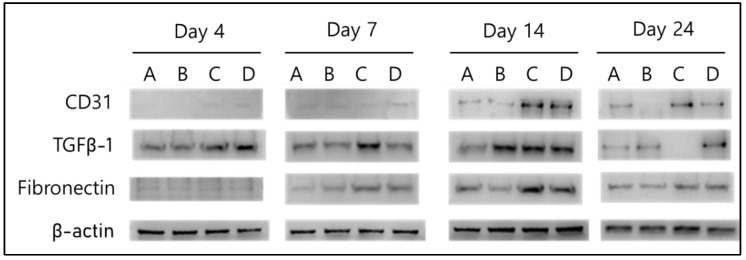
The expression pattern of CD31, TGFβ-1, and fibronectin protein using Western blot analysis in each group: for A. Gauze, B. Mepilex Lite, C. 2.25 wt% alginate solution + 3.0 wt% CaCl_2_ solution, D. 2.5 wt% alginate solution + 3.0 wt% CaCl_2_ solution.

Western blotting results for CD31, TGF β-1, and fibronectin showed that their expression in group C was generally higher than that in the control groups (groups A and B). In the first 4 days after the initial burn, the expression level of TGF β-1, a marker for wound healing, in groups C and D showed an increase of more than 10% compared to the control groups. Moreover, the expression of TGF β-1 increased the most in group C, and the expression of CD31, a renal vascular indicator, increased significantly by more than 10% in groups C and D compared to the control groups on day 14. The expression of fibronectin, an extracellular matrix formation marker, increased by more than 10% throughout the healing process in groups C and D compared to the control groups (Figure 6).

## 3. Conclusions

The skin is composed of two layers: the epidermis and the dermis. The epidermis acts as a barrier against bacterial invasion and moisture loss and can rebuild shallow wounds; however, in the dermis (part of the dermis or the entire dermis layer), which contains skin appendages, such as hair follicles, oil glands, and sweat glands, incomplete regeneration is inevitable after damage [23,24]. Studies are currently being conducted to achieve complete skin regeneration, as it remains an ongoing goal [25]. Currently, most ointments used to treat burns, normal wounds, and skin ulcers contain antibiotics and immunosuppressants. These ointments are simply applied to the skin to protect the skin tissue from excessive moisture loss, disease-causing microbial invasion, and harmful chemicals and to suppress secondary infections, inflammatory reactions, or only symptoms. Therefore, natural healing is expected. However, this is not an active treatment. For skin diseases such as burns and wounds, treatments with natural healing properties that can minimize scarring and effectively maintain skin regeneration are urgently required [26,27]. Burns are classified as severe depending on the extent of the burn area. Burns covering approximately 20% of the body surface area in adults and over 10% in children are considered severe. The causes of burns can be classified into thermal burns, chemical burns, inhalation burns, and others. Thermal burns refer to burns caused by fire, hot water, high-temperature liquid, or steam. Chemical burns refer to burns caused by contact with chemical substances, while inhalation burns occur when a person is damaged by inhaling high-temperature heat, carbon dioxide, or combustion substances during a fire in a closed space. First aid varies depending on the type of burn, but usually, when a burn occurs, cooling of the affected area is the most important factor that must be administered first. Additionally, in severe burns, early emergency burn treatment is paramount because the risk of life-threatening complications increases. The purpose of the dressing is to serve as a cover to protect the wound area and to support or fix the wound area by covering it with sterilized gauze or bandages. These dressings prevent further damage by suppressing bleeding and preventing infection at the wound site. The ideal conditions for an appropriate dressing should include the following factors: a moist environment, infection prevention, warming effect, protective action, moisture permeability, non-active, slight acidity, and the prevention of wound adhesion. However, there are limitations to their application in curved areas. In particular, when applied to the hands and feet, there is a possibility of adhesion between affected parts; therefore, the area between the fingers and toes must be dressed separately, which requires considerable effort. Because of the high costs incurred, more innovative burn-dressing methods are required. Currently, the interest in hydrogels, among other dressings for wound and burn treatment, is increasing. They reduce heat pain in the burn area and treat the wounds using a hydrogel fused with natural products such as tea tree oil. Researchers have reported that they prevent infections at burn sites [28,29,30,31]. However, they cannot form a physical barrier to prevent adhesion, relieve pain, or promote wound healing [32]. In addition, two biopolymers, gelatin methacryloyl and methacryloyl-substituted recombinant human tropoelastin, were manufactured through light-induced cross-linking to suppress infection, promote the healing of chronic wounds, and reduce the number of dressings. Even when applied to large, chronic, and difficult-to-heal wounds, it exhibited excellent adhesion and antibacterial properties, providing an alternative that can reduce the number of dressing changes [33,34,35]. However, despite these characteristics, there are still limitations in its application to various parts of the body, especially curved and wide areas. In this study, we used alginic acid, a natural polymer, to manufacture a safe, non-irritating spray-type hydrogel that can be widely used by people of all ages. Sodium alginate, a sodium salt of alginic acid, is soluble in water and has the unique property of easily forming a gel in the presence of multivalent metal cations such as Ca^2+^, Cu^2+^, and Zn^2+^. Therefore, alginate can be easily processed into various shapes through its gelation properties. However, the simple addition of aqueous CaCl_2_ solution to sodium alginate solution results in a heterogeneous sol/gel mixture including insoluble precipitates, which is hardly fabricated to a construct with a desired shape. For this reason, we prepared the hydrogel using the cross-linking method. In spite of this, the hydrogel is not able to absorb exudate as much as the sponge because it is a water-containing wound-dressing material. However, the hydrogel can provide a moist wound-healing condition without absorbing exudate from a wound. It is well known that alginate is a biocompatible polymer, and the treatment of cells with alginate extract dressings had little negative influence on cell viability and proliferation [36]. Accordingly, the developed material is used to minimize scars, suppress secondary infection or inflammatory reactions, improve recovery in case of skin damage by using it early in an emergency, and manufacture a formulation that can be applied to curved areas and wide areas that are emerging as the biggest problem during burns. In order to realize the stability of the manufactured formulation, a functional container that can be supported was produced, and the possibility of its use as a spray-type hydrogel dressing agent was evaluated. As a result, container suitability was carried out for injection implementation, and it was confirmed that the container designed and used by the researchers could be easily discharged and sprayed to a wide range. It was also confirmed that the gel was maintained for more than 6 h in all groups under wet surface conditions, and a stable hydrogel membrane was formed. The 2.5 wt% alginate solution + 3.0 wt% CaCl_2_ solution group, which showed stable gel membrane results, was subjected to viscoelastic behavior using a rheometer, showing G’ > G’ in the entire frequency region, confirming the rheological behavior of a typical alginate hydrogel. The prepared spray-type hydrogel was applied to the burn wounds in rats, and its effect on wound healing was investigated. We visually confirmed that the wound healing and contraction rates were significantly higher until the 14th day after the initial burn compared to that in the control groups (Figure 3 and Figure 4). Histological analysis showed that the collagen was arranged more densely, and it was confirmed to be in the mature healing stage. Initially, neovascularization was observed, and at the time of complete healing, a thick and mature regenerative epithelium was also observed (Figure 5 and Figure 6). Biochemical analysis showed no significant difference between the experimental and control groups after the first 4 d; however, it was observed that the expressions of CD31, TGF β-1, and fibronectin were slightly higher in groups C and D than in the control groups (A and B) (Figure 6). This means that wound healing is relatively fast, the epithelial basilar membrane is thick, and neovascularization occurs rapidly. Our findings show that alginate hydrogel is more effective in healing burns than conventional treatments and could be used clinically in the future. In the future, drugs effective in wound healing (*Centella asiatica*, sodium citrate, and tea tree oil) and pain relief local anesthetics (lidocaine, ropivacaine, and bupivacaine) can be combined to create a rapid and active emergency burn treatment material. They can also be applied to support skin regeneration and various medical device materials.

## 4. Materials and Methods

### 4.1. Preparation of Reagents

The materials used in the experiment were sodium alginate (LVM, 20–200 mPa·s, Nova Matrix, Sandvik, Norway), calcium chloride (CaCl_2_, Sigma-Aldrich, Saint Louis, MO, USA), ethyl alcohol (EtOH, Duk-san, Seoul, Republic of Korea), NaOH (Sigma-Aldrich, Saint Louis, MO, USA), and distilled water (DW, MiliQ quality, Billerica, MA, USA). All the other reagents used were of reagent grade.

**Figure 7:**
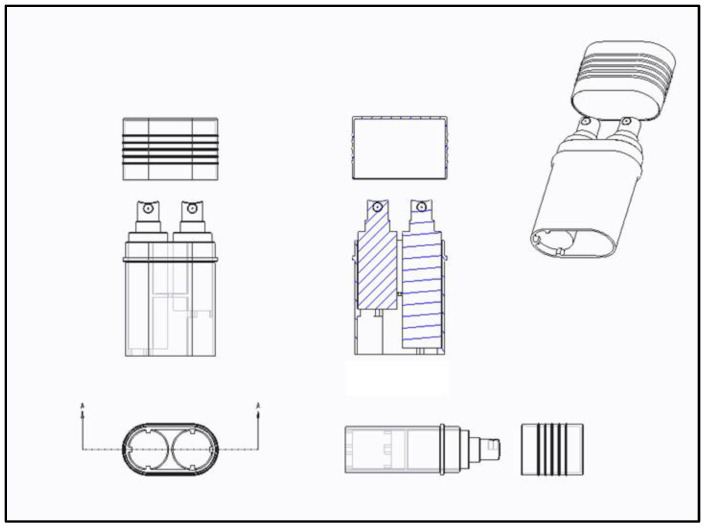
Design the container image to contain the spray dressing solution.

Briefly, 2.25, 2.5, and 3.0 wt% sodium alginate was added to the sterilized glass bottle and stirred at 80 rpm for 12 h with a stirrer to prepare an alginate solution. After that, it was sterilized with autoclave (JSAT-45, JS Research Inc., Seoul, Republic of Korea) at 121 °C for 15 min, and then the alginate solution was put into the manufactured spray container (Figure 7) using a sterilized bottle dispenser. After dissolving CaCl_2_ in DW at a ratio of 3 wt%, cross-linking solution was prepared by sterilizing and filtering using a bottle top filter (0.22 μm). Afterward, a spray container (Figure 7) was prepared using a sterilized bottle dispenser and used in the experiment for each group. To evaluate the optimal injection implementation at the manufactured alginate hydrogel concentration, the injection implementation was analyzed by designing a medical device that can provide drawings and MSDS data for medical device approval, as shown in Figure 7. It was designed in CAD, and then 3D printer (PLUS200, Seoul, Republic of Korea) produced it by printing. Afterward, spray dressing solution for burns was contained and evaluated for spraying implementation.

### 4.2. Analysis of Stability of a Gel Formulation

The gelation time was determined using the ball drop method [37]. First, to ensure homogeneity, 2.25, 2.5, and 3.0 wt% alginate solutions were added to test tubes and placed in a shaking water bath at 37 °C (SB-12L Shaking Water Bath, Benchmark Scientific, Inc., Sayreville, NH, USA) for 50 rpm before the experiment was conducted. In the manufactured compressed sprayer (Figure 7), 2.25, 2.5, and 3.0 wt% alginate solutions and 3.0 wt% CaCl_2_ solution were placed, respectively, and directly sprayed onto a steel square tray to check the gelation retention time. A steel square tray was judged to be in a gelled state when it was tilted at an angle of 30 degrees and stopped or remained within about 1.0 cm, depending on whether it was flowing. To determine the gelation time, pH 7.4 phosphate-buffered saline (PBS, Thermo Fisher Scientific Inc., Waltham, MA, USA) was sprayed at 30 min intervals to confirm whether the gel was maintained in a wet surface condition, and it was defined as the time from the moment of spraying to the gelation point.

To analyze the sprayed gelation stability according to the viscosity of the manufactured material, the gelation strength and retention time were measured on a Peltier adjusted to 25 °C using a rheometer (HAAKE™ MARS™, Thermo Fisher Scientific, Karlsruhe, Germany), and all measurements were repeated thrice.

### 4.3. In Vivo Model

**Figure 8:**
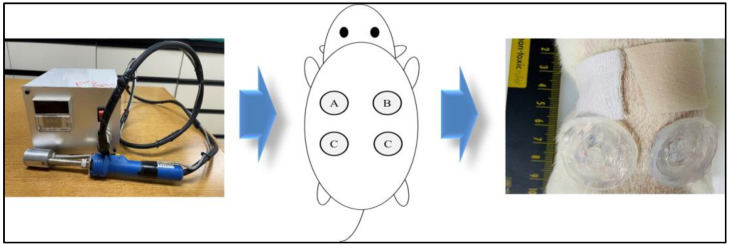
In vivo wound-healing test. Burn wounds were created by aluminum dermal block (130 °C, 5 s). The experimental group was divided into 4 groups: A. Gauze, B. Mepilex Lite, C. 2.25 wt% alginate solution with 3.0 wt% CaCl_2_ solution, D. 2.5 wt% alginate solution with 3.0 wt% CaCl_2_ solution. By applying the position of each group differently, the variable according to the position of the wound was minimized.

The ethical criteria of the Institutional Animal Care and Use Committee of Kyungpook National University Medical Center were followed. Forty male Sprague Dawley (SD) rats were used in this study. The rats were anesthetized before surgery through inhalation of Forane (Isoflurane; AErrane^®^, Baxter Healthcare Corporation, Deerfield, IL, USA). Subsequently, the mice were disinfected with betadine and alcohol. For the in vivo wound-healing test, burn wounds were created using an aluminum dermal block (130 °C for 5 s). Four round, deep, second-degree burn wounds (diameter of 2.5 cm) were made bilaterally on the dorsum of the rat’s trunk. The rats were then divided into four groups in which different dressing materials were applied: group A, gauze; group B, Mepilex™; group C, 2.25 wt% alginate solution with 3.0 wt% CaCl_2_ solution (Figure 8); group D, 2.5 wt% alginate solution with 3.0 wt% CaCl_2_ solution. By applying the position of each group differently, the variables were minimized according to the position of the wound. The gross findings of each group were compared by tracing the remaining wounds with imaging software and performing histological and biochemical analyses at each time point.

### 4.4. Visual Analysis

To confirm the visual healing evaluation of the SD rats, the healing area was measured using Image J1.47 software(National Institutes of Health, Bethesda, MD, USA) and the wound’s healing rate was calculated over time. The border of the entire layer window layer immediately after surgery was modeled on a sterilized transparent film, and the area was set as W_o_ (initial wound area, mm × mm). The area modeled by combining the epithelial parental tissue in the center of the window on a specific day and the area healed by the epithelialization around it was W_i_ (wound area on the days measure, mm × mm). The area of the non-epithelial parental tissue was U_i_ (area of un epithelized granulation tissue on the day measured). Transparent films were traced on days 4, 7, 14, and 24 after wound onset, digital photographs were taken, transparent films were drawn, and the total area of the window and epithelial window was calculated. Based on these values, the percentages of wound continuation (1), epithelialization (2), and healing (3) were calculated. 
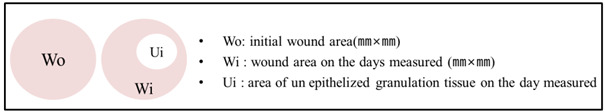

(1) $$\mathrm{Percentage}\;\mathrm{of}\;\mathrm{wound}\;\mathrm{contraction}=\frac{\left(\mathrm{Wo}-\mathrm{Wi}\right)}{\mathrm{Wo}}\times 100$$

(2) $$\mathrm{Percentage}\;\mathrm{of}\;\mathrm{wound}\;\mathrm{epithelization}=\frac{\left(\mathrm{Wi}-\mathrm{Ui}\right)}{\mathrm{Wo}}\times 100$$

(3) $$\mathrm{Percentage}\;\mathrm{of}\;\mathrm{wound}\;\mathrm{healed}=\frac{\left(\mathrm{Wo}-\mathrm{Ui}\right)}{\mathrm{Wo}}\times 100$$

### 4.5. Histological Analysis

For histological evaluation, the entire image, including some of the wound tissue around the burn area on days 4, 7, 14, and 24, was extracted after photographs were taken, placed in 10% formalin, fixed with paraffin, and sliced into tissue fragments and stained with hematoxylin & eosin (H&E) and Masson’s trichrome to observe the inflammatory response of the burn area, angiogenesis, formation and arrangement of collagen fibers, and epithelial regeneration under a light microscope (Olympus, Tokyo, Japan).

### 4.6. Biochemical Analysis

For biochemical evaluation, the wound tissue around the burn area was sampled on days 4, 7, 14, and 24, and the levels of proteins CD31 (PA5-32310), TGF β-1 (ab215715), and Fibronectin (ab268020), which are related to wound healing, were analyzed using Western blotting. The tissue was placed in a lysis buffer solution (Invitrogen, Carlsbad, CA, USA), then ground and pulverized using a homogenizer and stirred at 4 °C for approximately 30 min to completely dissolve. After measuring the protein concentration of the dissolving agent, an equal amount of protein was dissolved in the loading buffer, fractionated by loading onto a 12% SDS polyacrylamide gel (Invitrogen, Carlsbad, CA, USA), and transferred to a nitrocellulose membrane (Amersham™ Protran^®^, Leiden, The Netherlands). The nitrocellulose membrane was incubated with antibody solutions (Santa Cruz Biotechnology, Dallas, TX, USA) at a concentration of 1:200 for 2 h at room temperature and then washed. Subsequently, the anti-rabbit lgG antibody (Sigma, St. Louis, MO, USA) complexed with peroxidase at a dilution of 1:500 was used as the secondary antibody and incubated at room temperature for 1 h. After the reaction, the nitrocellulose membrane was subjected to protein expression and quantitative analysis using Image J1.47 with an extended chemiluminescent (ECL) system (Version 11.0, Medical Biology, Amsterdam UMC, Amsterdam, The Netherlands).

### 4.7. Statistical Analysis

All data were expressed as the mean ± standard error of the mean. All collected data were analyzed using *t*-tests and one-way analysis of variance. Difference-type data were collected and digitized using Microsoft Excel 2010.

## Data Availability

The data presented in this study are available on request from the corresponding author. The data are not publicly available due to ethical reasons.
